# Nesidioblastosis in the Adult Surgical Management

**DOI:** 10.1155/1997/81239

**Published:** 1997

**Authors:** J. Palla Garcia, Teresa França, Consiglieri Pedroso, Carlos Cardoso, M'Olímpia Cid

**Affiliations:** St. Martha's Hospital - General Surgery and Histopathology Wards Rua de Santa Marta Lisboa 1100 Portugal

## Abstract

Nesidioblastosis is an exceedingly rare occurrence in the adult and, when it appears, it is usually part of a MEA1 syndrome.

We present a case of nesidioblastosis in a young woman, with no concurrent endocrine pathology, while we discuss in detail the diagnostic and therapeutic problems posed by this condition.

The selected treatment was sub-total pancreatectomy (70–80%) and the histopathologic and immunohistochemical tests of the surgical specimen showed: “Diffuse Nesidioblastosis”.

The histopathologic and immuno-histochemical features of the resected pancreas are analysed in detail.


*HPB Surgery*, 1997, Vol.10, pp. 201–209
Reprints available directly from the publisher
Photocopying permitted by license only

© 1997 OPA (Overseas Publishers Association)
Amsterdam B.V. Published in The Netherlands
by Harwood Academic Publishers
Printed in India

### Nesidioblastosis in the Adult Surgical Management

J. PALLA GARCIA, TERESA FRANÇA, CONSIGLIERI PEDROSO, CARLOS CARDOSO and MªOLÍMPIA CID

*St. Martha's Hospital - General Surgery and Histopathology Wards (Rua de Santa Marta, 1100 Lisboa, Portugal)*

*(Received 21 July 1995)*

**Nesidioblastosis is an exceedingly rare occurrence in the adult and, when it appears, it is usually part of a MEA1 syndrome.**
**We present a case of nesidioblastosis in a young woman, with no concurrent endocrine pathology, while we discuss in detail the diagnostic and therapeutic problems posed by this condition.**
**The selected treatment was sub-total pancreatectomy (70–80%) and the histopathologic and immunohistochemical tests of the surgical specimen showed: "Diffuse Nesidioblastosis".**
**The histopathologic and immuno-histochemical features of the resected pancreas are analysed in detail.**

*Keywords:* Nesidioblastosis, pancreatectomy, Hyperinsulinism/hypoglycaemia

### INTRODUCTION

In the adult, the syndrome involving hypoglycaemic crisis associated with primary hyperinsulinism, immediately evokes the possibility of an insulinoma however, 10–15% of the adult patients with autonomous hyperinsulinism will have diffuse lesions to the islets, rather than a solitary adenoma[1]. In these situations of hyperinsulinism with diffuse lesions, in which the external appearance of the pancreas, as well as the imaging techniques, will not show any changes, the experience and skill of the histopathologist will constitute a major factor to the recognition and diagnosis of the actual lesions.

Three types of diffuse changes of the islet cells are known, and they may appear either at random, or by genetic determinism: islet cell adenomatosis, islet cell hyperplasia and nesidioblastosis[2].

The word "nesidioblastosis" comes from the Greek "νεσιδιυ" (nesidiu), which means "islet", and was originally proposed by Leidlow[3] and used by Vance[4] in reference to a diffuse lesion of the islets.

It is worth noting that nesidioblastosis is a developmental phase of the foetal pancreas and not an autonomous histopathologic entity, an impairment of the insulin storage and release capabilities being the best explanation for the hyperinsulinism found in these patients[5].

It's also worth mentioning that nesidioblastosis in the adult is an extraordinarily rare occurrence and, when one is found, it's usually part of the MEA syndrome.

### MATERIALS AND METHODS

### Clinical Case

We report a thirty years old female caucasian patient, with a history of hypoglycaemic crisis. She first presented in 1987 with bouts of intense sudoresis, tremor, loss of consciousness and transient right hemiparesis. She was diagnosed as having hypoglycaemic crisis, with a blood glucose of 2.2 mmol/L (normal 3.9–6.1)

Later she had two similar bouts, with similar hypoglycaemia.

In May 1990 she had an inconclusive fasting test, but subsequently had two more crisis of serious hypoglycaemia with loss of consciousness.

In January 1991 she was admitted to the General Surgery Department of St. Martha's Hospital, for a fasting test, which was inconclusive except for the very high levels of insulin. In May 1991 she was re-admitted, repeating the fasting test, which showed irregular changes in glycaemia, insulinaemia and the corrected ratio of the two variables.

Previous personal history and physical examination were non-contributors. From the available data we diagnosed a probable insulinoma. Following this diagnosis we performed some imaging of the pancreas, aimed at identifying the insulinoma, but this was inconclusive.

In January 1991 an abdominal CAT-scan showed "discreet nodularity of the pancreas tail": abdominal CAT-scan of May 1991 revealed that "the nodularity of the pancreas tail is still present". NMR-imaging of the pancreas showed no changes.

At angiography of the celiac branch (May 1991), showed a "tenuous venous flush in the transition area between the pancreas body and tail".

In spite of the problems posed by this lack of a sure pre-surgical identification of the insulinoma, we decided to proceed with surgery, with both diagnostic and therapeutic purposes. We believed that the patient had a small and solitary insulinoma, which we had to precisely localise and resect, its precise localisation being the first major surgical difficulty. With a complete and optimal exposure we went on to its systematic exploration, by visual inspection, careful palpation and pre-operative ultrasonography, we did not find an insulinoma.

In the absence of a detected insulinoma, but with a sure diagnosis of hypoglycaemia with autonomous hyperinsulinism, the possibility of nesidioblastosis was considered. In this condition, usually, the pancreas; appears normal and microscopic examination is not capable of defining this pathological process.

The decision to perform pancreatectomy in this case was based mainly on the clinical and laboratory features, and not on the surgical findings. We performed a sub-total pancreatectomy of 70% and splenectomy.

The sub-total pancreatectomy, measuring 7 × 3.5 cm, was serially sectioned, with an average thickness of 0.5 cm. The cut surface of the slices showed the usual appearance of pancreatic lobulation and no tumours were seen.

A large number of paraffin sections were examined these under optical microscopy stained by Haema-toxilin-Eosin. Some of the slices were observed with histochemical techniques, by the Grimelius method, and also immuno-histochemically by the immunoperoxydasis method, various hormonal anti-bodies were used: insulin ($\beta$ cells), glucagon ($\alpha$ cells), somatostatin ($\delta$ cells), VIP ($\delta$ cells), Substance-P, Bombesine, Gastrine and Chromogranine, as an anti-body for endocrine cells.

### RESULTS

### Histology

Pancreatic parenchyma had no changes in the exocrine tissue, but the endocrine tissue was both morphologically and topographically altered, with variation in the size and number of islets, with loss of their normal centro-lobular distribution.

With immuno-histochemical staining for insulin an unusual diffuse proliferation of marker-positive cells, was seen with a random distribution within the pancreatic lobule, either isolated, or in small aggregates of 2 or more cells (Fig. 1a, b).

Insulin-positive cells were also seen, not only in the epithelium of pancreatic ducts, but alsoin their proximity, in the form of cell conglomerates,

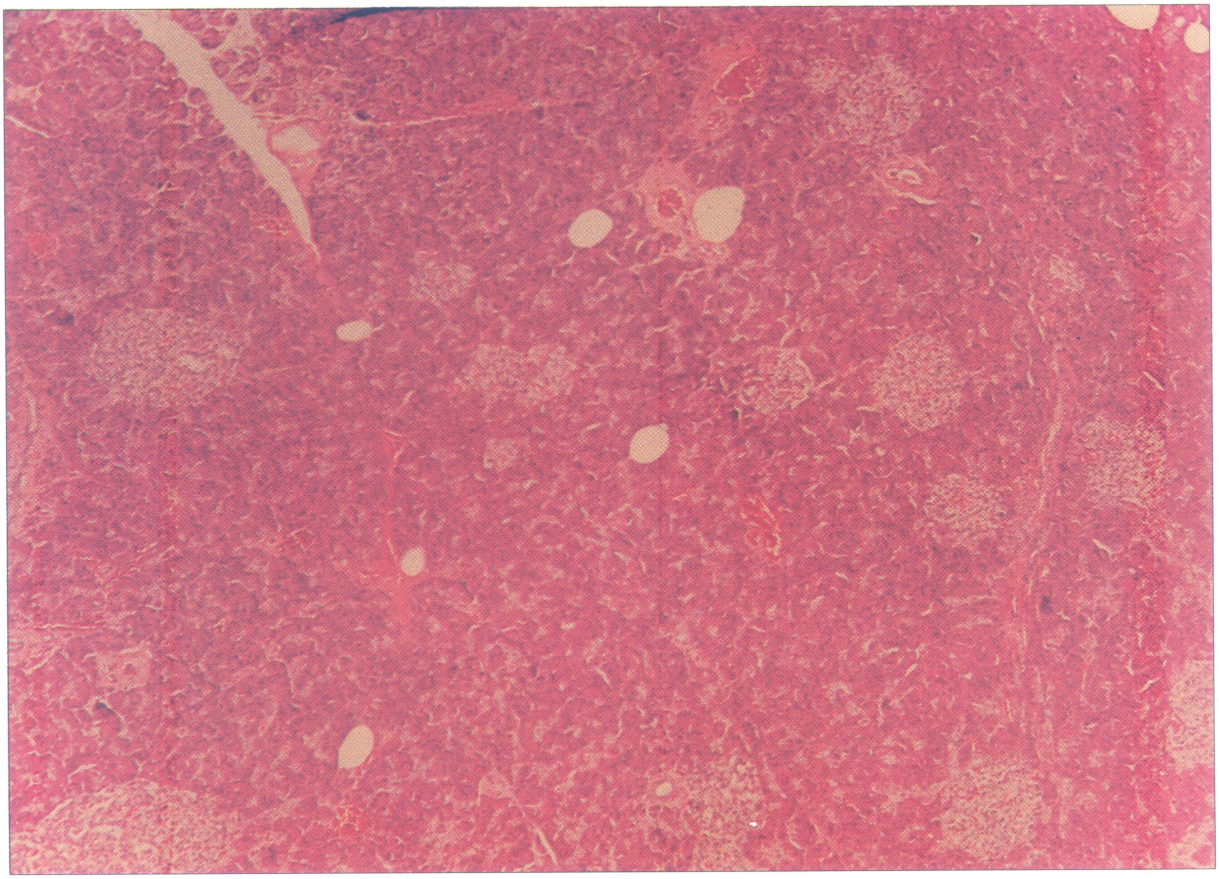

FIGURE 1A Diffuse proliferation of small endocrine cell conglomerates and islets. H.E., ×40. (See Color Plate I).

sometimes multiple, but often as duct-endocrine proliferations (Fig. 2). An enormous size is reached by some islets which also have an irregular shape. There is occasional nuclear variability, both in size and staining (Fig. 3).

The hormonal tests, besides insulin, included other hormonal markers for which we established a ranking between one (+) and four (++++) proportional to the number of cells that had cytoplasm positive to each marker. A negative immuno-marker response was assumed in the absence of brown cytoplasmic staining. The following results were thus obtained:

Insulin (β cells) ............ (++++)

Glucagon (α cells) ....... (+++)

Somatostatin (δ cells) . (+++)

VIP (δ2 cells) ................ (++)

Bombesine .................... (−)

Gastrine ........................ (−)

Chromogranine ........... (++++)

The immediate post-operative period was without event. Four months later she had a abdominal CAT-scan (September 1991) which showed "post surgical status, with normal topographic organ arrangement".

However, during the first year post-surgery, the patient had two new crisis of hypoglycaemia with blood glucose about 2 mmol/L. Glycaemia and insulinaemia levels between crisis were normal. It was decided to start medical therapy with infused long-acting somatostatin, for periods of 5 days, with good results during 1992/93, during which she had no more crisis. Early in 1994, two and a half years after the pancreatic resection, she received a long acting somatostatin, Sandostatin® (octreotide) sub-cutaneously, 100 μg t.i.d., as an outpatient. At the end of 1995 – four years after operation she was quite well, without further hypoglycaemic crisis.

### DISCUSSION

Nesidioblastosis is the main cause of hyperinsulinism/hypoglycaemia in new-borns and children, being a very rare condition in adults. In these, as in adolescents, the main cause of hyperinsulinism/hypoglycaemia is the presence of a pancreatic adenoma of insulin-producing cells. Until 1981 nesidioblastosis in adults[6] had always been described in association with other diseases, such as: Zollinger-Ellison syndrome, multiple endocrine adenomatosis, congenital neuroblastoma, Lindau's disease, chronic pancreatitis and cysticfibrosis. In the last 12 years a very small number of cases of nesidioblastosis in the adult, unassociated with other diseases, has been described in the literature.

It was George F. Leidlow[3] who, for the first time in 1938, defined the morphological lesion of nesidioblastosis as a "diffuse or disseminated proliferation of the islet cells.... originating in the ductules or pancreatic ducts"[6]. Later, Yakovac (1986) wrote that: "nesidioblastosis consists of insulin-producing cells, either isolated, or in

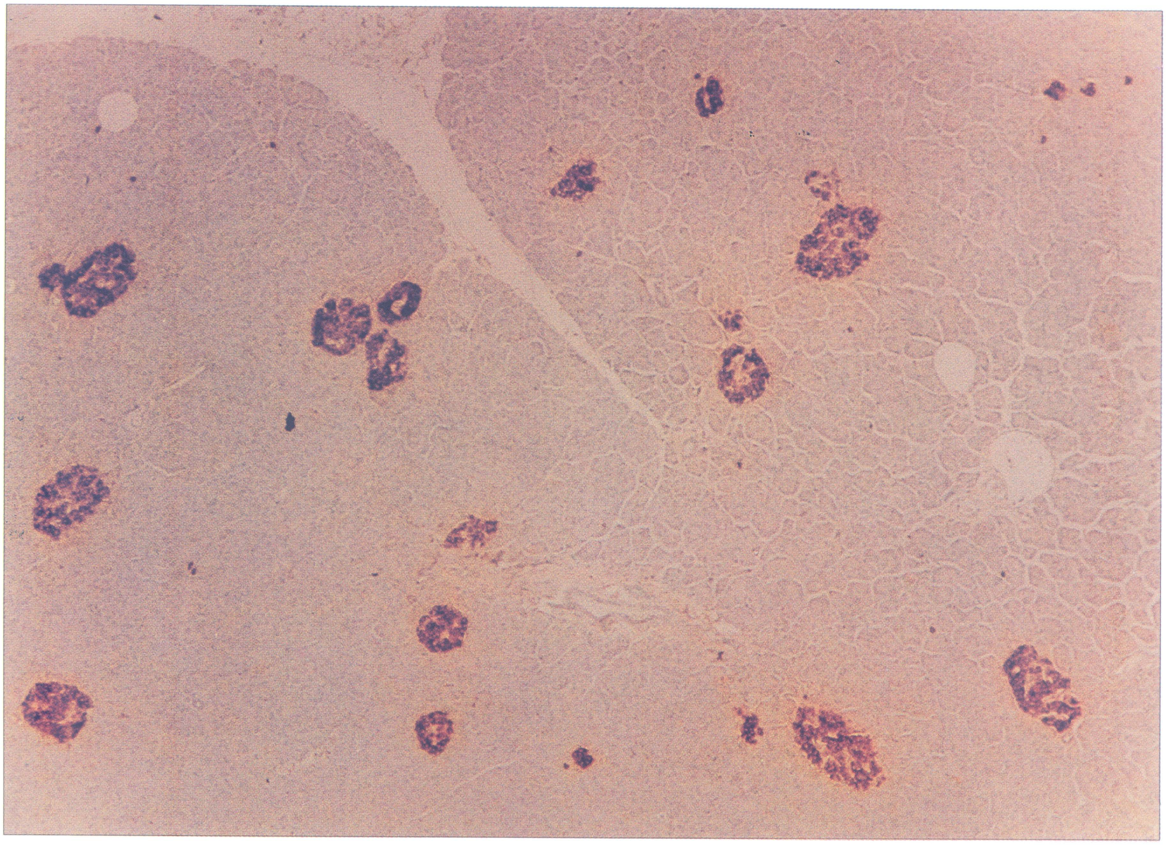

FIGURE 1B Minuscule cell aggregates enhanced by the insulin-sensitive immuno-peroxydasis technique, ×40. (See Color Plate II).

small groups of 2–6 cells, dispersed throughout the parenchyma, often in relation to the epithelium of exocrine ducts"[7]. These morphological changes, with distortion of the pancreatic lobular architecture, due to endocrine cell proliferation, had been previously mentioned by some authors under the name of nesidiodysplasia[6]. Later this designation was attributed to other morphological criteria: increase in nuclear diameter and staining, in islet endocrine cells[8]. In our case all these morphological criteria, distortion of the lobular architecture, endocrine duct/ductule proliferation, changes in size and irregularity of the islets were present and we also found discret occasional changes in nuclear diameter and staining, i.e., β cell hypertrophy.

This disease of the Langerhans Islets of the pancreas, represents an entity difficult to treat, and difficult to understand. The awkward histologic appearance of nesidioblastosis, chains of cells along the pancreatic ducts while real islets are not to be found, has lead some authors to consider these lesions as a block to islet differentiation during foetal life. But with foetal development and islet embryogenesis better understanding due to advances in microscopy and biochemistry, it became obvious that nesidioblastosis, hyperplasia of the islet cells and adenomatosis, are phases in the foetal development of the pancreas and represent different aspects of a common condition, resulting from the incomplete islet maturation.

The symptomatology present in these rare situations resutls from the intermittent and variable insulin hypersecretion. The pathophysiological relationship between the structural changes of the islets and hyperinsulinism is as yet unclear. Four mechanisms have been proposed[5] to explain hyperinsulinism in interest hypoglycaemia associated with nesidioblastosis: a) increase in the number of cells within the islets; b) relative increase in the number of β cells and a reduction of the glucagon and somatostatin secreting cells; c) changes in islet architecture resulting in impairment of their para-endocrine function; d) changes in the functional regulation of β cells impairing the storage and release of insulin.

The increase in the mass of islet cells associated with the anatomical findings of adenomatosis, hyperplasia and nesidioblastosis, mentioned by various authors[9,10,11] has not been confirmed, however, using histometry parameters[12,13,14].

The relative increase of β cells with reduction of the glucagon and somatostatin secreting cells[15,16] has also not been found. Depsite abnormal islet architecture and impairment of the para-endocrine function having been referred to as a possible mechanism for hyper insulinism[17] this has also been found in normal controls. The impairment of storage and release of insulin remains the possible mechanism for the clinical manifestation.

The fact that the correction of hypoglycaemia is only temporary, with recurring bouts of hypoglycaemia in patients subjected to 70% pancreatic resection may be explained because such resections have reduced the total mass of islet cells, but left behind cells that apparently still have a flaw in the regulation of insulin production, and are capable of hypersecretion with the resulting hypoglycaemia.

Near-total pancreatectomy frequently seems to be necessary and, even in some of these patients submitted to an extensive pancreatic resectoin and left only with a small number of remaining islets, the incidence of Diabetes mellitus is surprisingly low.

In spite of the best surgical treatment not yet having been completely established neither dietary measuresnor insulin suppressing drugs have shown an efficacy comparable to resection surgery. Total pancreatectomy will, however, always lead to endocrine and exocrine impairment, which may be badly tolerated by children and adolescents.

Early trends in the management of this condition were relatively conservative, limiting the extent of the resection to 70–80% (sub-total pancreatectomy); however the frequent recurrence of hypoglycaemia, mainly in infants and small children, made re-operations frequent, and there is now a marked trend towards near-total pancreatectomy – 95%.

Scott-Bjerk[17] says that, in his experience of babies with 70–80% pancreatic resection, all had to be reoperated with 90–95% resection to control hypoglycaemia. With older children he had better results with 70–80% resection, but they required post-surgery adjunct therapy.

Kramer[18] found more extensive structural lesions of the islets in the younger patients, consisting of a massive increase of endocrine cells, with absence of normal secretion by this endocrine tissue. In older children and adults the structural changes in the islets were more subtle and islet irregularity, with mixed endocrine and exocrine tissues, only became obvious with special histochemical techniques and more thorough searching. However all of Norton's work[19,20,21] underlines the need for near-total pancreatectomy (95%), preserving the spleen, as the definitive treatment of nesidioblastosis in the child, this guideline to be followed from early childhood, even when a normal looking pancreas is found.

In the adult, due to the great rarity of this condition, there are no available data in the literature to allow for any conclusions to be drawn; we should, however, think from its late onset that the islet lesions should be less hyperfunctioning and, that in the adult having all its

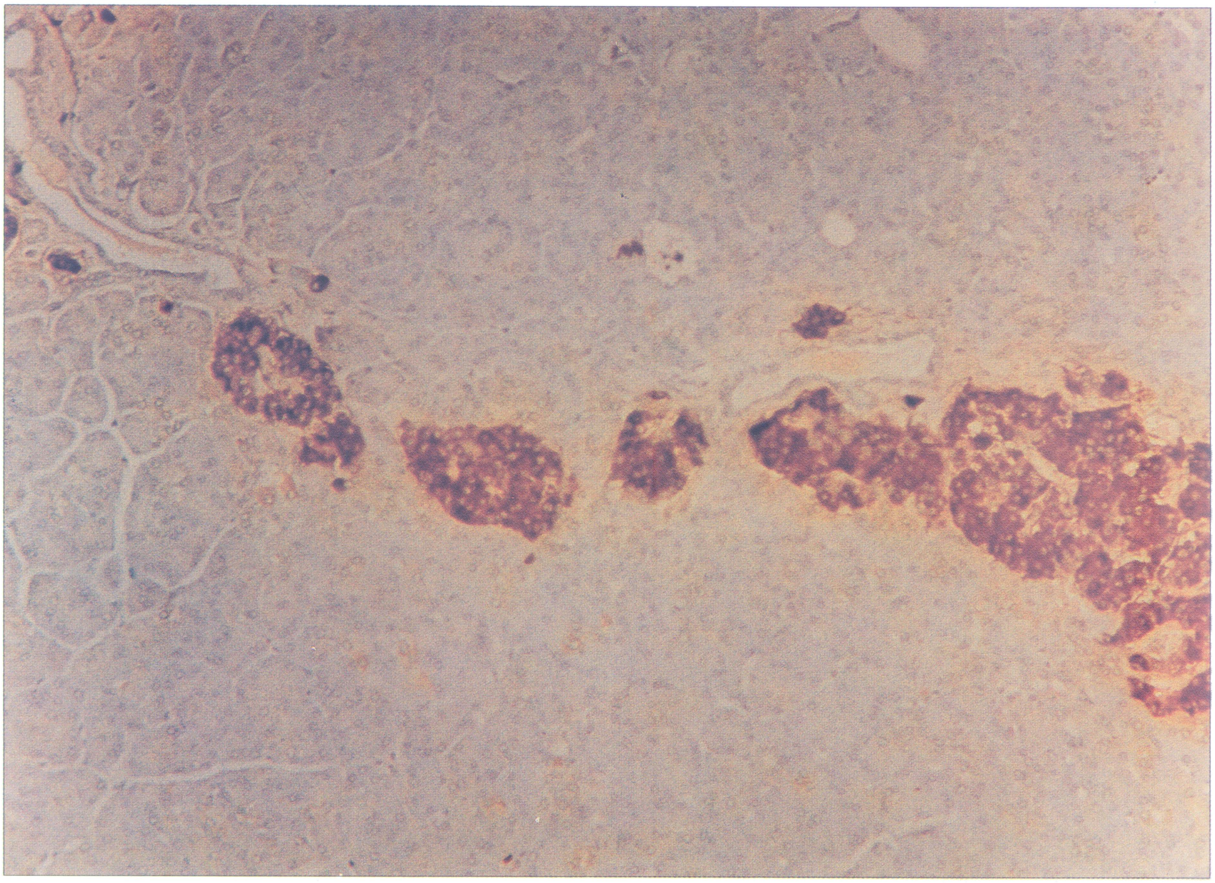

FIGURE 2 Duct-islet proliferation. Insulin-sensitive immuno-peroxydasis technique, ×100. (See Color Plate III).

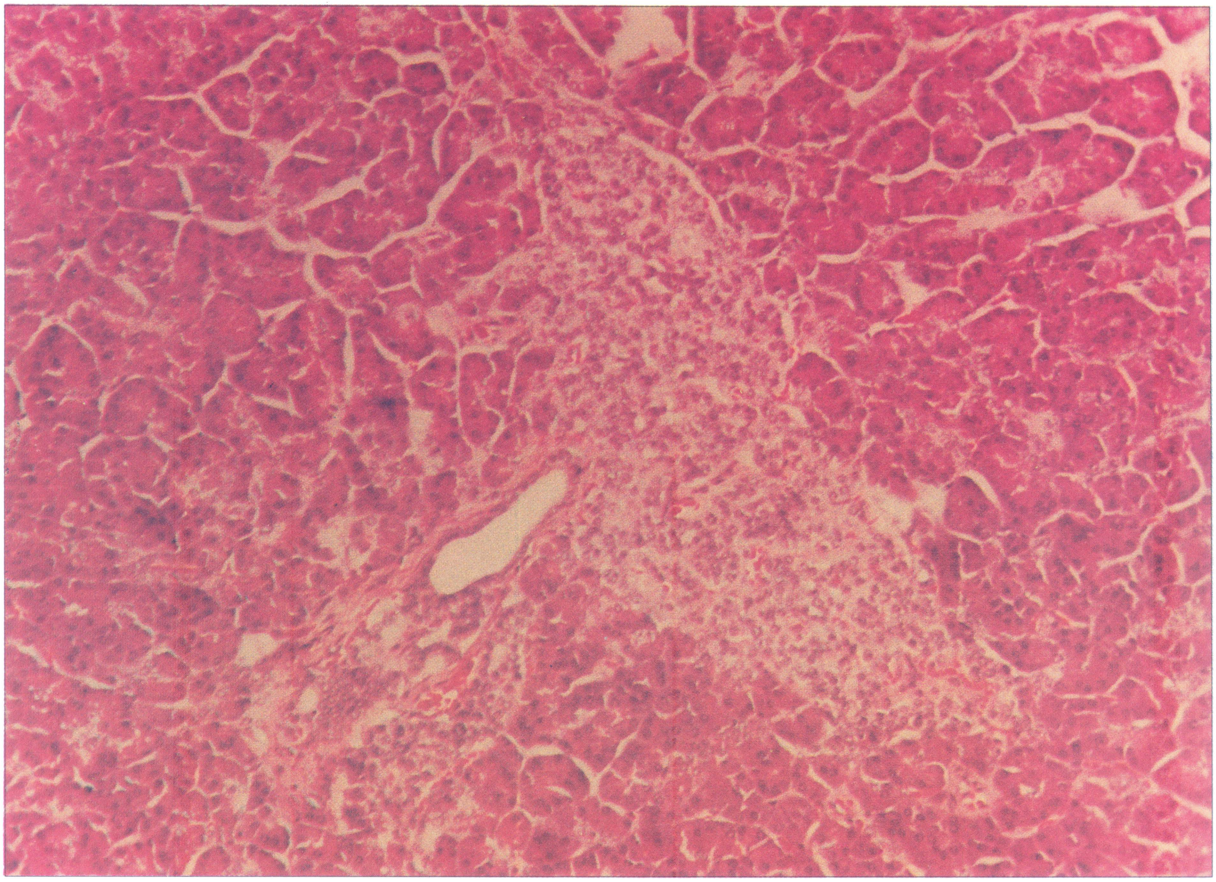

FIGURE 3A Duct-islet proliferation with very large and irregular Langerhan islet, H.E., ×100. (See Color Plate IV).

systems fully developed, the consequences of hypoglycaemia would be more attenuated.

It should be noted, however, that the current management in the child, and possibly in the adult, is the need for an early diagnosis and early aggressive surgical treatment, with extensive resection, saving the patient from the risks and complications of recurring hypoglycaemia.

Our patient, with a sub-total 70% pancreatectomy, is still under follow-up therapy and has had, in the 5 following years, 4 milder bouts of hypoglycaemia, apparently controlled and prevented by therapy with octreotide.

Long-acting somatostatin, which is described as having an inhibitory action on the release of insulin, growth-hormone, glucagon, gastrin and other peptides, would have a palliative effect and, although a decrease in the growth of endocrine tumours has been shown experimentally in the rat, its effects are currently still under evaluation.

Clinically octeotride has been shown to be effective in the management of hypoglycaemia in children with nesidioblastosis, although it has not been as effective in adults with insulinoma[22,23]. Occasional hypoglycaemia control in a few patients with long term octeotride has also been mentioned by some investigators[24].

We recognise the need to keep our patient under close follow-up and, if hypoglycaemia bouts recur, we will have to re-evaluate her and plan a possible reoperation for near-total pancreatectomy.

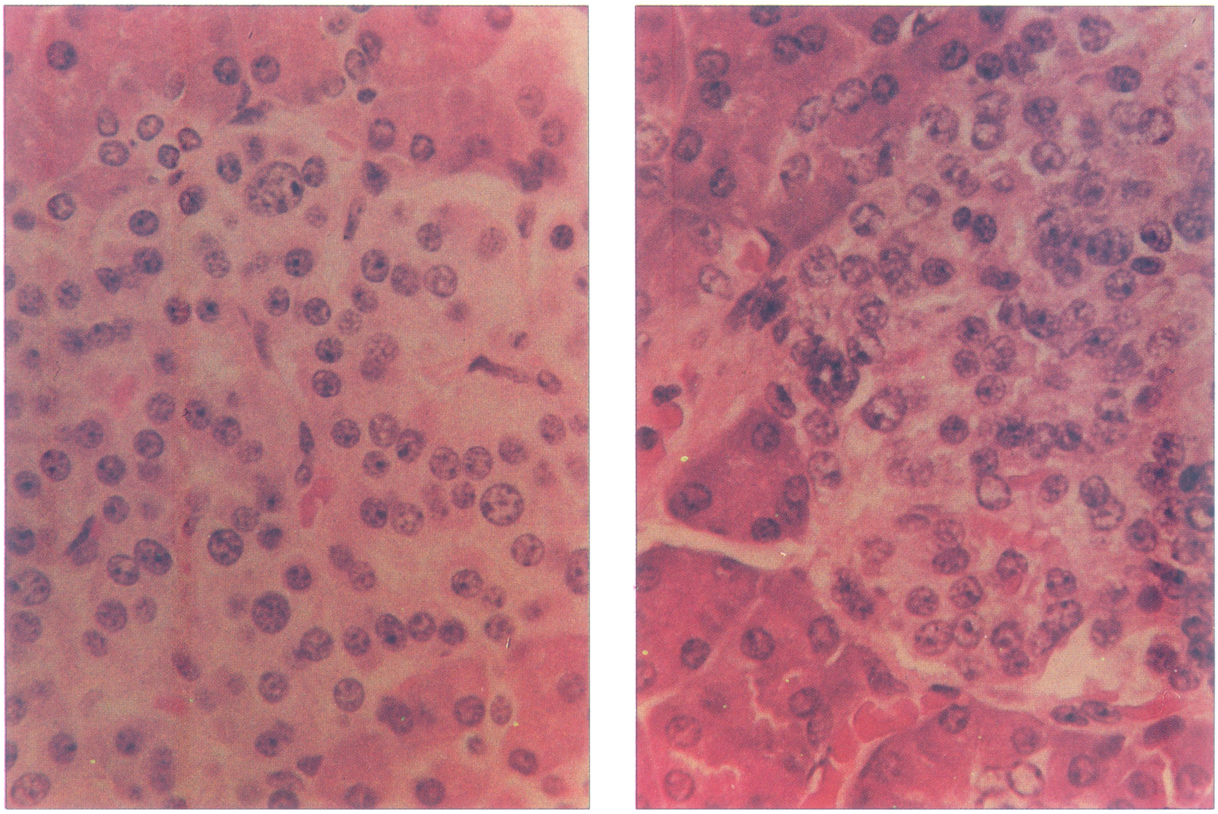

FIGURE 3B, C Nuclear changes in size and staining H.E., ×400. (See Color Plate V).

### References

[1] Keivunun, D.G. and Harrison T. (1990). The Hypoglicemic Syndrome: Endogenous Hyperinsulinismo in: Surgical Endocrinology Clinical-Syndrome Stanley Friesen Norman Tompson pàg. **223**, Editor J.B. Lippincett. Comp.

[2] Friesen (1990). Editorial Comment in: Surgical Endocrinology Clinical Syndromes by Stand Friesen Norman Tompson pàg. 229, Editor J.B. Lippincett Comp.

[3] Laidlaw G.F. (1938). Nesidioblastoma the islect tumeur of the pancreas. *Am. J. Pathology*, **14**, 125.

[4] Vance J.E. (1972). Familiar nesidioblastosis as the predominant manifestation of the multiple endocrine adenomatosis. *Am. J. Med.*, **52**, 211.

[5] Thomas, G.G., Guenco. R.E., Azigklin, R.G. and Underward L.E. (1988). Changing concepts of islect cell dysplasia in neonatal and infantile hyperinsulinism. *World J. Surg.*, **12**, 598.

[6] Jay, K. Harness *et al.* (Nesidioblastosisin adults) 1981. (May) *Arch. Surg*, **116**, 575.

[7] Goudswaard *et al* (1986). (Nesidioblastosis and Endocrine Hyperplasia of the Pancreas: A secondary Phenomenon) *Human Pathology*, **17**, 46–53.

[8] I. Ariel. al. (Nesidiodysplasia. A histologic Entity?) *Human Pathology*, **19**, 1215–1218 by Sauders Company.

[9] Heitz, R.V., Klappel, G., Hacki, W.H., Polack, J.M. and Pearse A.C. (1977). Nesidioblastosis: the pathologic basis of persistent hyperinsulinémia hypoglicémia in infants. *Diabetes*, **26**, 632.

[10] Shermetta, D.W., Mendelsh Haller (1980). Hyperinsulinemia hypoglycémia of the neonate associated with persistent fetal histology and function of pancreas. *Ann. Surg.*, **191**, 182.

[11] Gouldl, V.E., Memeli, V.A., Dardi, L.E. and Goudel N.S. (1981). Nesidiodysplasia and nesidioblastosis of infancy: Ultrastructural and immunohistochemical analysis of islet cell alterations with and without associated hyperinsulinemic hipoglycaemic. *Scand J. Gastroenterolog.*, **70**, 129.

[12] White, A.P., Greider, M.H. and DE Sehrgver k. kissane (1990). The juvenile human endocrine pancreas: Normal versus idiopatic hypereinsulinemic hypoglycemia. *Semin. Diag. Patholog*, **1**, 130.

[13] Jaffe, R., Hashide, Y. and Yunis E. (1986). Pancreatic pathology in hiperinsulinemic hypoglycemia of infancy. *Lab. Invest*, **42**, 356.

[14] Simmons, P.S., Talander, R.L., Carney, J.A., Walel, L.E. and Haymond M.W. (1984). Surgical management of hyperinsulinemic hypoglycemia in children. *Arch. Surg.*, **119**, 520.

[15] Bishop, A.E., Polak, J.M., Chese, P.G., Timsen, C.M.,

Bryant, M.G. and Bloom S.R. (1981). Decrease of pancreatic somatostatin in neonatal nesidioblastosis. *Diabetes*, **30**, 122.

[16] Falkmer, S. Svik, O. and Vidnes J. (1981). Immunohistochemical, morphometric and clinical studies of the pancreatic islects in enfants with persistent neonatal hypoglycemia. *Acta Bol. Med. Gerieat*, **40**, 39.

[17] H. Scott Bjerka *et al.* (1990). Oct (Surgical Management of islect cell Dysmaturation syndrome in young children).

[18] Kramer, J. Martin Bell, De Schryver, Richard Bower, Jessie Ternberg and Neil Withe (1982). Clinical and Hystologic indications for extensive pancreatic resection in Nesidioblastosis - *Am. J. Surg*. 143–116–8

[19] Norton. Doppman and Jansen (1989). Cancer of the endocrine system In de Vito Cancer: principles and practice of oncology Phila delphia Lippincott. 1269–1344.

[20] Bolande R.P. (1974). The neurocrestopathioes: a unyfrying concept of disease arising from neural crest maldevelopment Human. *Pathol.*, **5**, 409.

[21] Pearse (1980). The APUD concept and hormone production. *Clin. Endocrinol. Metab.*, **9**, 211.

[22] Maton R.M. (1989). The use of the long acting somatostatin analogue, octeotride in patients with islect cell tumuers Gastroenterology. *Clin. N.A.*, **18**, 897–922.

[23] Glaser, B., Rosler, A. and Halperin Y. (1990). Chronic treatment of benign insulinoma using the long-acting somatostatin analogues *SMS Isr. J. Med.*, **26**, 16–9.

[24] Laron Z. *et al.* (1990). Somatostatin analogues in the management of benign insulinomas. *Isr. J. Med.*, **26**, 1–2.

